# A wearable biochemical sensor for monitoring alcohol consumption lifestyle through Ethyl glucuronide (EtG) detection in human sweat

**DOI:** 10.1038/srep23111

**Published:** 2016-03-21

**Authors:** Anjan Panneer Selvam, Sriram Muthukumar, Vikramshankar Kamakoti, Shalini Prasad

**Affiliations:** 1 Department of Bioengineering, 800 W. Campbell Rd, University of Texas at Dallas, TX 75080; 2 EnLiSense LLC, 1813 Audubon Pond way, Allen, TX 75013, USA.

## Abstract

We demonstrate for the first time a wearable biochemical sensor for monitoring alcohol consumption through the detection and quantification of a metabolite of ethanol, ethyl glucuronide (EtG). We designed and fabricated two co-planar sensors with gold and zinc oxide as sensing electrodes. We also designed a LED based reporting for the presence of EtG in the human sweat samples. The sensor functions on affinity based immunoassay principles whereby monoclonal antibodies for EtG were immobilized on the electrodes using thiol based chemistry. Detection of EtG from human sweat was achieved through chemiresistive sensing mechanism. In this method, an AC voltage was applied across the two coplanar electrodes and the impedance across the sensor electrodes was measured and calibrated for physiologically relevant doses of EtG in human sweat. EtG detection over a dose concentration of 0.001–100 μg/L was demonstrated on both glass and polyimide substrates. Detection sensitivity was lower at 1 μg/L with gold electrodes as compared to ZnO, which had detection sensitivity of 0.001 μg/L. Based on the detection range the wearable sensor has the ability to detect alcohol consumption of up to 11 standard drinks in the US over a period of 4 to 9 hours.

Alcohol (ethanol) is the most abused drug worldwide. Alcohol abuse affects over 18.5 million adults in the U.S., costing society over $200 billion annually in lost productivity, health care expenditures, motor vehicle accidents, crime and other related costs^1^. Alcohol related diseases contribute to a high percentage of all hospital admissions (>20%) and deaths ~100,000 annually^2^,^3^. Hence, there is an imminent and unmet need for the development of effective diagnostic tests to detect high-risk drinking behavior and alcohol-induced tissue damage. The measurement of ethanol in blood itself is the most objective method for quickly confirming its presence in an individual of interest. However, the low specificity and very short window of detection renders ethanol itself as a less reliable marker for diagnosing and monitoring long-term physiological and behavior effects. Ethanol has an additional disadvantage that it can also be detected in the sugar containing body fluids of diabetics exposed to bacteria making it not suitable marker for body fluids other than blood. The ideal marker should be sensitive and specific as well as have a long window of detection in a number of body fluids not limited to blood. The emergence of non-oxidative direct metabolites of ethanol biochemical markers Ethylglucuronide (EtG), Ethyl Sulfate (EtS) for the detection of alcohol has grown in recent years. The desire for such biochemical markers as objective measures for evaluating new medications or behavioral interventions for alcohol problems has prompted the recent surge in the study and testing of EtG to meet these requirements^4^,^5^,^6^,^7^,^8^,^9^,^10^.

Typically, it has been observed that 0.7% of the ethanol consumed is excreted through the breath, constituting that measured by a breath alcohol detector. However, an additional 0.1% of the ethanol consumed is transported to the surface of the skin where it exits the body through sweat^11^,^12^,^13^. Additional disposition pathways of ethanol in blood involves oxidation by the cytosolic enzymes, alcohol dehydrogenase and aldehyde dehydrogenase and to a much lesser extent microsomal CYP2E1^14^. Recently, ethyl glucuronide (EtG) and more recently ethyl sulfate (EtS), minor metabolites of ethanol produced by conjugation pathways, have been investigated as markers of ethanol exposure and their pharmacokinetics well characterized^15^,^16^,^17^,^18^,^19^. EtG is a minor metabolite of alcohol that forms in the liver when alcohol reacts with glucuronic acid, a substance that works to detoxify drugs by turning them into water-soluble compounds that are then easily removed from the body and detectable in body fluids such as sweat, urine, and hair^20^,^21^,^22^,^23^,^24^. The chemical pathway for EtG metabolism is shown in Fig. 1(c). Although comprising only about 0.1% or less of ethanol’s total disposition, EtG can usually be detected for 24 or more hours after one or two “drinks”, and for as long as two to four days after heavier consumption. This makes EtG especially useful for detecting drinking relapses and is an ideal marker for zero tolerance as well as abstinence. Currently the accepted testing methodologies for the detection of EtG in serum/plasma or urine utilize Immunoassays (EIA and ELISA), gas chromatography and mass spectrometry. Assays are not suitable for on-site or point of care diagnostics and does not allow for real time feedback to patients as might be optimal for contingency management interventions. Hence, there is an opportunity to monitor alcohol consumption through monitoring for EtG in a temporal manner from human sweat using wearable biosensors.

The field of wearable sensors has developed substantially over the past decade, with most solutions targeting healthcare applications via the transduction of physical parameters such as heart rate, respiration rate, blood oxygen content, skin temperature, bodily motion, brain activity, and blood pressure^25^,^26^,^27^. The need to bridge the gap between powerful analytical devices and user requirements of convenience, comfort, and small size while providing simplicity of operation, flexibility, and timely presentation of results is imperative in such applications and can all be addressed with current developments in the electrochemical-based wearable sensors domain. In this paper, we utilized gold and zinc oxide as electrode sensing elements in designing a flexible biosensor suitable for sweat based detection of EtG.

Gold (Au) has been widely used for affinity based electrochemical biosensing mechanisms due to its stability in body fluids and the ability to leverage gold-thiol interactions for immobilizing target biomolecules on to gold surfaces. Gold is widely used in electrical and electrochemical detection of biomolecules leveraging charge transfer for detecting and quantifying biomolecule concentrations. Current commercially available test strips for blood glucose sensing are electrochemical-based using functionalized gold electrodes^28^. Metal oxide-based nanomaterials with high isoelectric point (IEP) are suitable for the immobilization of biomolecules with low IEP via electrostatic interactions. Among the various metal oxide material systems, Zinc oxide (ZnO) has necessary characteristics that make it an excellent candidate material for wearable biosensing applications. These include excellent biocompatibility to both the wearer and in preserving the stability of the bound biomolecules, high isoelectric point (pH ≈ 9.5), favorable linker chemistries and fast electron-charge transfer, ease in formation of nanostructures, and a heightened sensitivity to adsorbed molecules. Aside from the inherent polarity in the ZnO crystal, the tetrahedral coordination is an indicator of sp^3^ covalent bonding. However, the Zn–O bond also possesses very strong ionic character, and thus ZnO lies on the borderline between being classed as a covalent and ionic compound, with an ionicity of f_i_ = 0.616 on the Phillips iconicity^29^. Surfaces such as these are beneficial for sensing of charged biomolecules such as EtG because they can improve charge-transfer based molecular binding on select surfaces with high specificity as well as enhanced sensitivity of the bound molecules.

Flexible substrates such as polyimide (Kapton), polyethylene naphthalate (PEN), polyethylene terephthalate (PET), polytetrafluoroethylene (Teflon), among others have long been employed in the printed electronics industry^30^,^31^,^32^. Owing to their intrinsic plasticity, hydrophobicity, excellent dielectric properties, thermal stability, low coefficient of thermal expansion, structural resiliency against repeated bending forces, and compatibility with roll-to-roll fabrication processes for low cost and scalable manufacturing; these flexible substrates have served as the platform of choice in various printed circuit board assemblies^33^. Recently, electrochemical devices that leverage these materials for the fabrication of thin-film sensors aim at coupling the merits of flexible substrates listed above along with their electrochemically inert nature^31^,^34^,^35^,^36^.

In this paper, we demonstrate the detection of EtG in human sweat using label-free electrochemical chemi-impedance sensing method, towards designing a flexible and wearable sensor prototype. We have incorporated single capture immunoassay sensing methodology onto a conformal electronic surface of polyimide that enables chemiresistive signal transduction. We utilized gold and zinc oxide electrodes integrated on flexible polyimide and compared the performance with rigid glass substrates as electrochemical sensors to perform detection and continuous monitoring of EtG from human sweat in a wearable configuration using chemiresistive mechanism of detection. We demonstrate the output response of this prototype chemiresistive EtG sensor using an optical light emitting diode based reporting. Figure 1(a–f) summarizes the sensing methodology, sensors presented in this study and a schematic representation of the optical readout system used. Figure 1(a) is the representation of sweat absorption onto sensing electrodes and binding events at the electrode-sweat interface. Figure 1(b) shows the chemical structure of the thiol linker used for immobilizing the capture antibodies on the electrodes. Figure 1(c) represents the chemical signaling pathways for metabolism of EtG, EtS and Acetic acid by the body post alcohol consumption. Figure 1(d) are optical images of the Au and ZnO sensors on glass and polyimide substrates – In clockwise order: ZnO electrode array on polyimide substrate with Au as an ohmic contact; ZnO on polyimide diced and sliced for testing with EtG doses; Au electrodes on glass substrate (post dicing) for EtG testing. Figure 1(e) is the optical LED display for classification of measured EtG concentration as low (Green LED), medium (yellow LED) and high (red LED) concentrations. Figure 1(f) shows the modified Randles equivalent circuit used for interpreting the impedance data for Au and ZnO electrodes.

## Results and Discussion

### Electrical characterization and noise signal estimation

Baseline sensor impedance was measured as 10 kΩ and 3.6 kΩ for Au electrodes on glass and polyimide substrates respectively and 28 kΩ and 4.7 kΩ for ZnO electrodes on glass and polyimide substrates respectively. A total of n = 3 replicates were performed and CV of 8% was observed. The measured impedance values indicate the resistive nature of the electrode-sensing regions and these values were used as control for all subsequent assay immobilization steps on these sensors. Impedance change was measured to characterize the chemiresistive interactions occurring on the sensor surface with assay immobilization steps. The parameters for this characterization was defined at 10 mV peak-to-peak voltage and at 10 Hz. Figure 2(a–d) shows the change in impedance for DSP functionalized sensor surface (i.e. after 1x PBS solution and treatment with 10 mM DSP for 1 hour) for Au and ZnO electrodes on both glass and polyimide substrates. Figure 2(a) shows the Au electrode sensor on glass substrate. Figure 2(b) shows the ZnO electrode sensor on glass substrate. Figure 2(c) shows the Au electrode sensor on flexible substrate and Fig. 2(d) shows the ZnO sensor on flexible substrate. Each assay step showed statistically significant impedance change (marked with asterisk and connected with brackets) with threshold set at 0.05 (p < 0.05). Evaluation of noise with PBS (P1–P3) and EtG free sweat (S1–S2) on Au electrode sensor on glass substrate and Au electrode sensor on flexible substrate, ZnO electrode sensor on glass substrate and ZnO electrode sensor on flexible substrate are shown in Fig. 2(e–h) respectively. Error bars represent standard deviation of the mean for n = 3 replicates.

The impedance increased from 10 kΩ to 480 kΩ ohms after DSP functionalization for the Au sensor on glass substrate while for the ZnO sensor on glass substrate, a change in impedance from 28 kΩ to 1134 kΩ was observed for DSP functionalization. This change is characteristic and is in good correlation with the resistive nature of DSP in DMSO solution. Similar impedance trend was seen for Au and ZnO sensor on flexible polyimide porous substrates. We performed a 3x DMSO wash post DSP functionalization to remove any unbound linker molecules, followed by a 3x PBS wash to prepare the sensor surface for immobilization of antibodies in this case anti-EtG. The impedance change for pre-antibody treatment to after 30 minutes EtG antibody incubation is also shown in Fig. 2(a–d). We did not notice any statistically significant change in impedance post 30 minutes of EtG antibody treatment. The impedance measured post EtG antibody treatment was 16 kΩ and 4.6 kΩ for Au electrodes on glass and polyimide substrates respectively and 41 kΩ and 6.9 kΩ for ZnO electrodes on glass and polyimide substrates respectively. The decrease in impedance from post DSP functionalization to post antibody immobilization is due to the increase in charge conducting molecules bound to the electrode surface forming an electrically charged double layer. We performed a noise signal estimation to quantify impedance changes due to background buffer and non-specific binding. The sensors were washed 5x times with PBS to remove unbound antibody molecules and impedance measurements was performed for further validation of the accuracy and stability of antibody binding. We did not find statistically significant differences post the first wash and after each wash indicating successful immobilization of EtG antibody and shown in Fig. 2(e–h). We tested the antibody-coated sensor for noise in PBS and human sweat (tested negative for EtG). The nominal impedance change for the anti-EtG, antibody immobilized sensor was 2.5 kΩ for the Au electrode and as 3.2 kΩ for the ZnO electrode on glass substrate while on polyimide substrate was 0.64 kΩ for the Au electrode and as 0.92 kΩ for the ZnO electrode. The drop in nominal impedance is characteristic and in good correlation with the dielectric nature of glass and polyimide substrates. In summary, both Au and ZnO electrodes demonstrate a stable and consistent impedance trends with assay functionalization indicating a robust binding of EtG antibody to the sensing surfaces on both rigid glass and flexible polyimide substrates.

### Impedance based characterization of EtG binding

**Figure 1:**
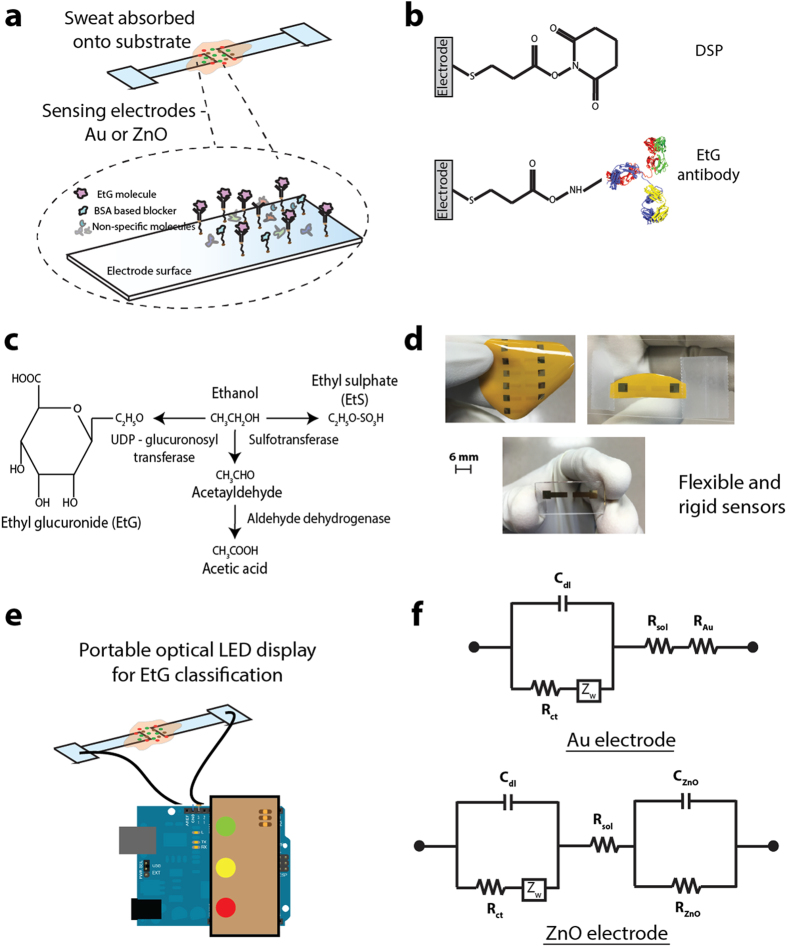
Schematic of integrated glass and flexible substrate based sensing platform for point and continuous detection. (**a**) Schematic of assay and molecular binding. (**b**) Antibody immobilization strategy. (**c**) EtG, EtS and Acetic acid signaling pathway post alcohol consumption. (**d**) Optical images of the Au and ZnO sensors on glass and polyimide substrates. (**e**) Optical LED display for classification of measured EtG concentration. (**f**) Modified Randles equivalent circuit for interpreting the impedance data from Au and ZnO electrodes.

**Figure 2:**
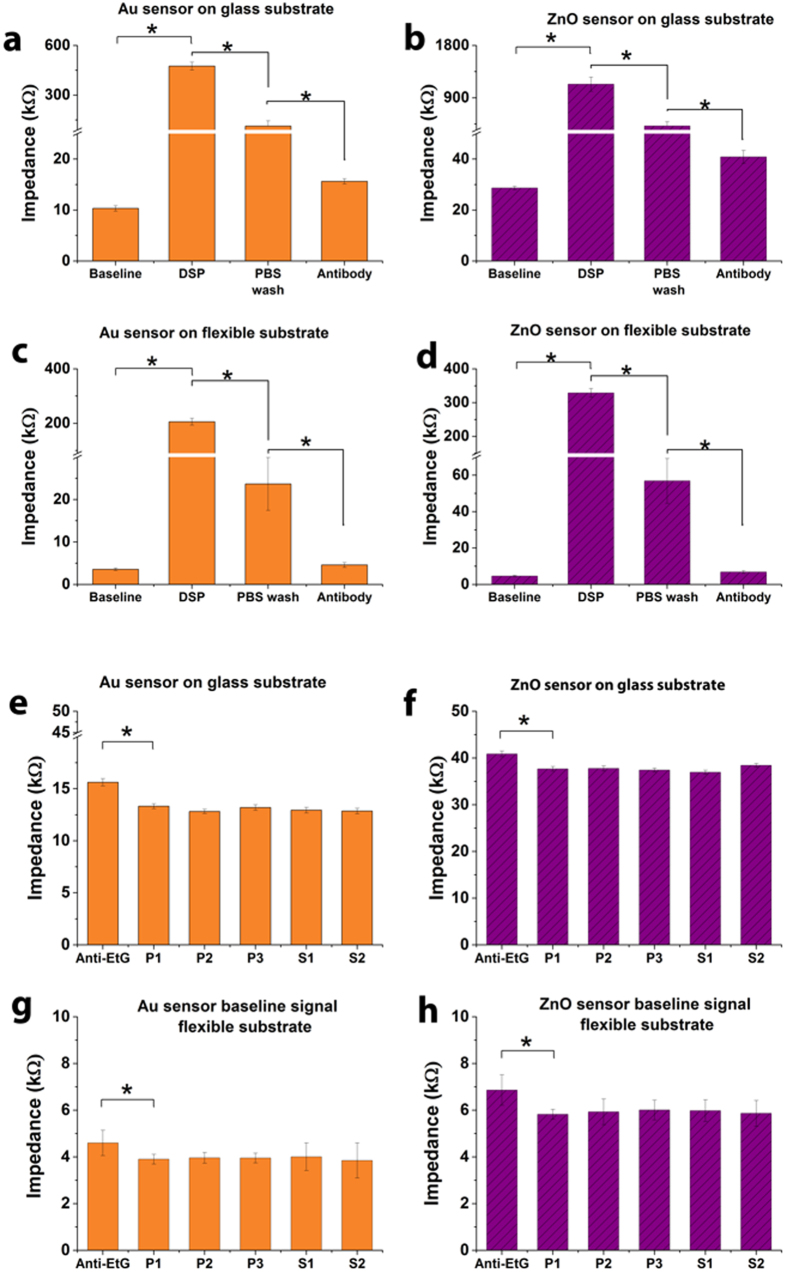
(**a–d**) Baseline (PBS), DSP functionalization and antibody immobilization with buffer steps as control. Each assay step showed statistically significant impedance change (marked with asterisk and connected with brackets) with threshold set at 0.05 (p < 0.05). (**e–h**) Evaluation of noise with PBS (P1 – P3) and EtG free sweat (S1 – S2). Error bars represent standard deviation of the mean for n = 3 replicates.

**Figure 3:**
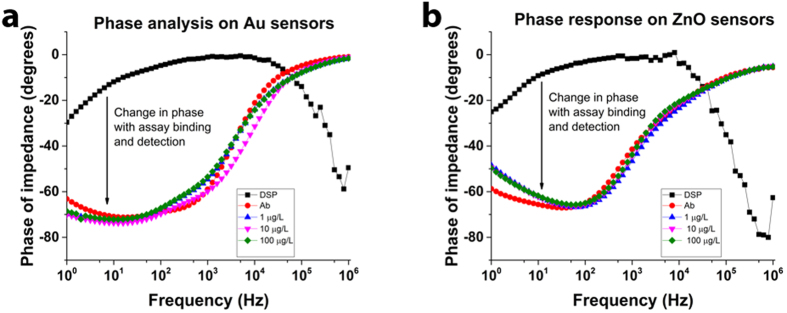
Impedance phase spectra to identify electrical nature of binding on the Au and ZnO sensor electrodes (**a**) Phase plot on Au sensors and (**b**) ZnO sensors after antibody immobilization and EtG detection. Data from this plot was collected from n = 3 replicates and fitted to the equivalent circuit model for identification of impedance dominating component.

We compared the phase plots of the impedance measured over a range of frequencies to characterize the nature of binding on the electrode surfaces shown in Fig. 3(a,b). A definitive lag in output response with respect to the applied voltage was observed indicating the formation of a capacitance dominated impedance element at the electrode surface. We observed a 60 degrees phase lag in the output impedance response indicating the capacitive nature of the sensor response. We used a modified Randles equivalent circuit, shown in Fig. 1(f) to study the nature of the impedance spectra. The measured impedance was identified to be dominated by the C_dl_, which is the capacitive of the double layer formed at the sensing electrode-fluid interface. We identified this observation to be in alignment with previously published literature by our group and many other researchers^37^,^38^,^39^,^40^,^41^. The formation of an affinity immunoassay on a conducting material i.e. Au surface in this case or semi-conducting material i.e. ZnO surface in this case, resulted in the polarization of charges on the sensing electrode surface and a complementing charge layer formed at a defined height offset from the surface in the fluid. This is in accordance with the Gouy chapman model for the formation of an electrical double layer at the electrode-fluid interface that forms a capacitive dominating impedance element at low frequencies of 10 Hz^42^,^43^.

### Dose response curve for quantification of EtG

**Figure 4:**
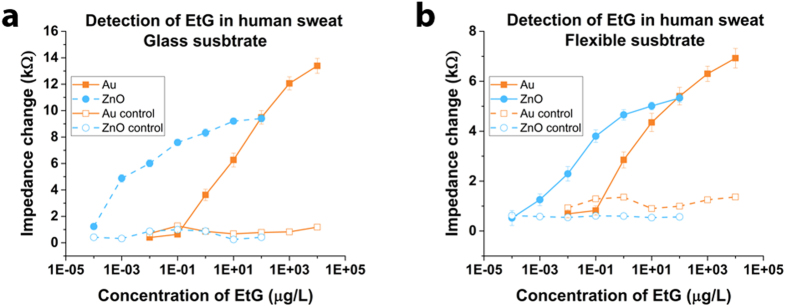
Dose response curve for calibrating sensor impedance output with EtG dose concentration in pooled human sweat. (**a**) Response curve for Au and ZnO sensors on glass and (**b**) Response curve for Au and ZnO sensors on flexible polyimide. Impedance change for normal sensor showed statistically significant changes with p threshold set at 0.05 (p < 0.05) when compared with the control (p > 0.05).

We tested different dose concentrations of EtG in the physiologically relevant range of 0.1 ng/L–10 mg/L spiked in 3–6 μL pooled human sweat on independent EtG antibody immobilized sensors to establish the impedance-concentration correlation for creating calibration dose response curves. Figure 4(a,b) shows the calibration response curves for the two electrode materials on both glass and polyimide substrates respectively. Specificity of detection and signal change was tested on Bovine Serum Albumin (BSA) coated sensor in the absence of the monoclonal EtG antibody. We tested the different concentrations of EtG spiked in human sweat and the observed response for the two electrodes are shown in Fig. 4(a,b). Au electrode sensors demonstrated detection of EtG with signal above the noise in the range of 1–10,000 μg/L on both substrates. The range of impedance change for Au electrode was 3.6 kΩ to 13.3 kΩ that accounted for a 73% dynamic range of signal on glass substrate and was 2.9 kΩ to 6.9 kΩ that accounted for a 59% dynamic range of signal on polyimide substrate. ZnO electrode sensors demonstrated detection of EtG with signal above the noise in the range of 0.001–100 μg/L on both substrates. The range of impedance change for ZnO electrode was 4.9 kΩ to 9.4 kΩ that accounted for a 48% dynamic range of signal on glass substrate and was 1.3 kΩ to 5.3 kΩ that accounted for a 76% dynamic range of signal on polyimide substrate. The error bars represent standard error over mean calculated from a total of n = 5 replicates. Limit of detection was identified as follows : Au on glass −1 μg/L ; ZnO on glass −0.0001 μg/L; Au on polyimide −1 μg/L; ZnO on polyimide −0.001 μg/L.

The published study reported to date on quantitative EtG measurements in sweat collected from human volunteers using a sweat patch (PharmChek) and analyzed using a mass spectrometer to range from 1.7 to 103.0 μg/L for alcohol consumption ranging from 38.0 to 154.6 grams equivalent of pure alcohol^44^. In the same study, zero EtG was detected in human subjects who did not consume alcohol i.e. abstinence cases. When compared to this published study, the range of EtG levels reported meets the capability of the sensor demonstrated using both Au and ZnO electrodes. According to the Centers for Disease Control and Prevention, USA, a standard drink in US is equal to 14.0 grams (0.6 ounces) of pure alcohol^45^. Generally, this amount of pure alcohol is found in 12-ounces of beer (5% alcohol content); 8-ounces of malt liquor (7% alcohol content); or 5-ounces of wine (12% alcohol content). At these levels of consumption (i.e. ≤ 38 grams of equivalent pure alcohol) or below, we estimate that the sensor using ZnO electrodes would be more suitable for reliably detecting very low EtG levels in human sweat.

### Continuous detection of EtG

**Figure 5:**
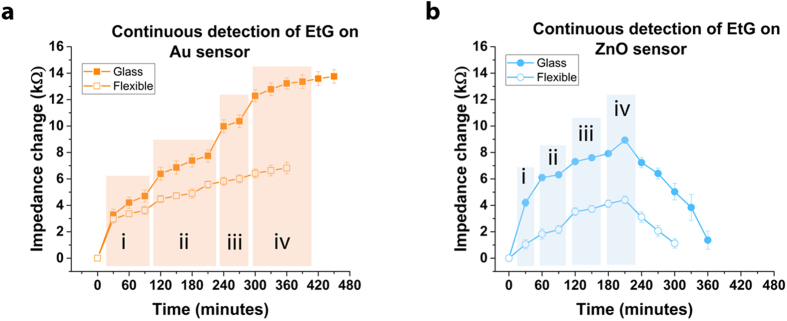
Continuous detection and monitoring of EtG in sweat on glass and flexible substrates. Impedance change as response for detection of EtG in human sweat introduced to the (**a**) Au electrode sensor surface tested with EtG at i –1 μg/L; ii –10 μg/L; iii –100 μg/L; iv −1000 μg/L; v −10000 μg/L and (**b**) ZnO electrode sensor surface with EtG at i –0.001 μg/L; ii –0.01 μg/L; iii –0.1 μg/L; iv −1 μg/L.

Detection of EtG when present in perspired human sweat requires continuous real-time monitoring of the user. We designed an experiment to evaluate the ability of the sensor device towards continuous real-time detection of EtG. To simulate real world testing, we performed this process at room temperature (25–28 deg C) and ambient relative humidity of ~65%. We introduced sweat spiked with EtG at increasing concentrations and at intervals of 30 minutes to the sensor surface and measured the impedance change at 10 Hz (2 minutes post interaction of sweat with electrode). Baseline impedance was measured at time t = 0 for the two electrodes. The impedance change was estimated with reference to the baseline as shown in Fig. 5(a–d) for Au and ZnO sensors on both glass and polyimide substrates. EtG in the concentration range of 1–10000 μg/L was tested on the Au sensors and in the concentration of 0.001–100 μg/L on the ZnO sensors. The EtG dose concentrations were increased logarithmically. Change in the EtG dose introduced to the sensor surface was performed when signal saturation was observed or when half the concentration of the next EtG test dose was reached. The detection response study was performed for up to a total time of 9 hours. The Au sensor showed a steady response for the total duration of the test providing a stable detection output for the sweat samples tested. The ZnO electrodes demonstrated stability in response for up to ~4 hours and afterwards showed drop in impedance. We hypothesize this trend of lower impedance change observed on the polar ZnO surface is due to the ionic interferents present in sweat.

### Sensor Performance Metrics

**Figure 6:**
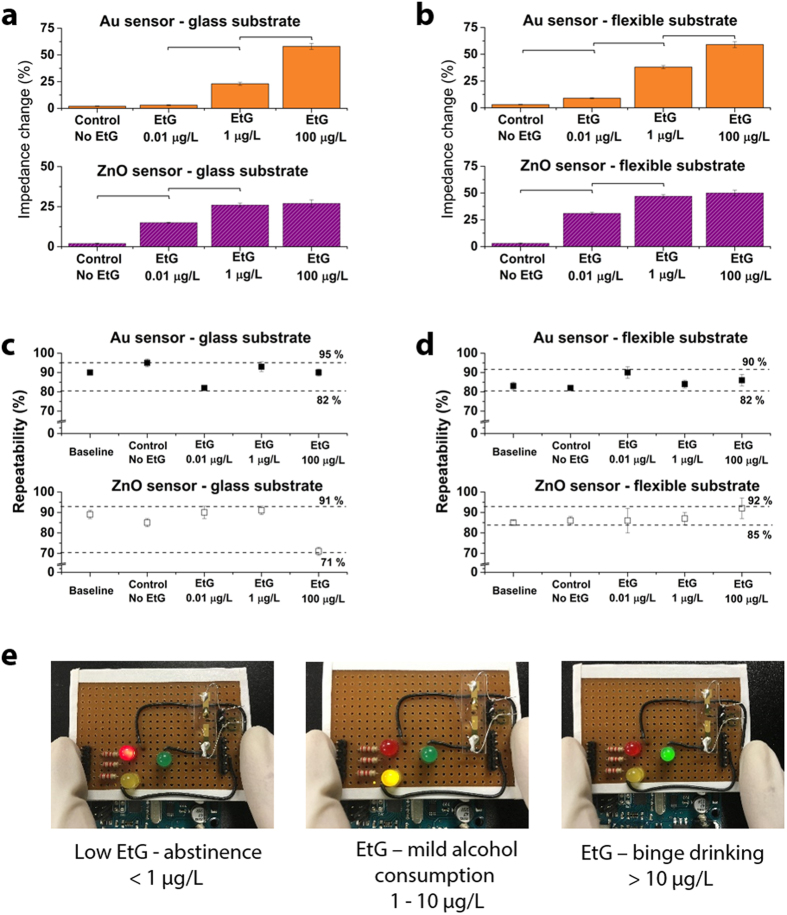
Evaluation of sensor performance and optical LED readout for user communication. **(a,b)** Sensor response to blind samples of EtG tested on Au and ZnO sensors. **(c,d)** Demonstrate repeatability of the system for the Au and ZnO sensors. **(e)** Smart optical LED readout display for classifying alcohol consumption behavior. Statistical significance threshold was set at 0.05 (p < 0.05). Error bars represent standard error of the mean form n = 3 replicates.

A sensor is required to demonstrate consistent and robust performance in a real world setting when tested with a multitude of samples. For example, sweat perspired varies in its constituents from person to person and hence comes with an inherent variability. This requires the sensor to be able to self-calibrate and demonstrate repeatability in performance. We performed an analysis of repeatability, sensitivity and selectivity by testing the Au and ZnO sensors on glass and flexible substrates with pooled sweat from different stock batches. The samples prepared for this study were blinded and tested for a total of n = 3 replicates by three different users. To evaluate the strength of the calibration curve, we used the dose response curve to estimate the concentration of EtG in the blinded samples was estimated from the measured impedance values by fitting it with the dose response curve. The percentage change in impedance observed for these samples is shown in Fig. 6(a–d). Linear regression analysis was performed on the measured impedance with dose response impedance and a R^2^ of 0.97 was observed for the Au and ZnO sensors. This demonstrated the selectivity and sensitivity of the sensor towards detection of EtG. The observed limit of detection for Au electrodes on glass and flexible polyimide was 1 μg/L. Recent advances in the field of gold electrode biosensors for flexible and rigid biosensors demonstrate sensitivity in the ranges of 0.1 μg/L–10 μg/L^46^,^47^,^48^. The observed sensor performance in our case is in this range of performance. In the case of ZnO electrodes on the glass and flexible substrates, we observed a detection limit of 0.0001 μg/L and 0.001 μg/L respectively. In comparison to most ZnO based biosensors which have limit of detection in the 0.01 μg/L to 1 μg/L^49^,^50^,^51^,^52^, we demonstrate a higher level of sensitivity. This is attributed to the ability to selectively probe the electrical double layer regions where analyte binding occurs, hence improving the signal to noise ration.

Statistical assessment was performed using ANOVA and p value of <0.05 was observed for the two electrode materials on the glass and flexible substrates in this study. The Au and ZnO sensor systems demonstrated a repeatability between 71% and 95% for the detection of EtG at concentration required for classification of abstinence, mild alcohol consumption and binge drinking.

### Portable display for EtG monitoring

In order to demonstrate the proof of feasibility of this sensor to function as a wearable non-invasive sensor that can provide information on the wearer’s alcohol use, we designed a simple electronic interface for reporting the senor output in a user-friendly format. A simple and communicable modality is essential when EtG found in perspired sweat crosses a certain concentration threshold especially in the case of abstinence practice (example: patients with liver related diseases, etc.). We designed and built a logic-based circuit to output an optical based signal upon EtG detection exceeding the threshold and to notify and provide a visual cue to the user from avoiding further alcohol consumption / dietary changes until the EtG levels have dropped below threshold. An ARM microcontroller based electronic reader as shown in Fig. 6 was setup to provide input voltage signal to the sensor as well as read the output current/impedance from the sensor. Figure 6(a) shows the sensor response to blind samples of EtG tested on Au and ZnO sensors on (a) glass substrate and (b) flexible substrate. The control used was an antibody-immobilized sensor tested with EtG free sweat. Figure 6(c,d) demonstrate repeatability of the system to be between 71% and 95% for the Au and ZnO sensors on the two substrate materials. Figure (e) shows the smart optical LED readout display for classifying alcohol consumption behavior.

Operational amplifiers at the sensor-microcontroller interface ensured low frequency noise isolation and AC signal smoothening. A current sense circuit enabled the sensitive detection of current from the sensor. Programmable logic was used to estimate impedance change and predict concentration of EtG present in the sweat on the sensor electrodes. We classified the range of EtG concentration as high, medium and safe for this study based on published studies^44^. Based on the estimated concentration (from measured impedance and pre-loaded dose response curve) of EtG, a LED based signal in terms of red, yellow and green was generated as shown in Fig. 6.

## Conclusions

In summary, we have demonstrated a flexible biochemical sensor platform suitable for wearable biosensing of EtG from human sweat. We have calibrated the sensor performance as a steady state assay as well as a continuous use assay. EtG is known to remain in the human body for up to 3 days after consumption of alcohol and therefore is useful for monitoring abstinence and identifying relapse to heavy drinking. We have demonstrated performance capabilities (sensitivity ranges, specificity, and stability) of an Au electrode based EtG sensors and a ZnO electrode based EtG sensors as a single-use and a continuous-use monitoring devices. Au electrode based EtG sensors with a capability to detect in concentration range of 1–10000 μg/L and up to ~9 hours are better suited for monitoring devices towards limiting alcohol consumption i.e. identifying relapse to heavy drinking. The observed limit of detection for the Au sensor on glass and polyimide was 1 μg/L which is comparable to detection limits observed in recently developed gold electrode based biosensors and wearable devices. ZnO electrode based EtG sensors with a capability to detect in concentration range of 0.001–100 μg/L and up to ~4 hours are better suited as abstinence monitoring devices. ZnO sensors demonstrated a limit of detection of 0.0001 μg/L on glass substrates and 0.001 μg/L on polyimide substrates. We demonstrate a better sensitivity with the use of ZnO electrodes when compared to most recent biosensor developments. This is primarily due to the presented novel method which captures the changes in the electrical double layer where binding occurs. An efficient sensor system for EtG detection would therefore be a combination of sensors fabricated using both Au electrodes and ZnO electrodes and configured in a lateral flow or multiplexed manner. We demonstrate this concept in this paper through a simple optical display using logic based approach to report EtG levels in human sweat.

## Experimental Section

### Materials

Polyimide substrates were procured from GE Healthcare Life Sciences (Piscataway, NJ, USA) with 0.2 μm pore size. Glass substrates were procured from Fisher Scientific Inc. (Waltham, MA, USA). The linker molecule Dithiobis [Succinimidyl Propionate] (DSP) and its solvent Dimethyl Sulfoxide (DMSO) were ordered from Thermo Fisher Scientific Inc. (Waltham, MA, USA). Antibody to EtG was purchased from EastCoast Bio (North Berwick, ME, USA). The antibody was purified monoclonal and stored at 4 °C. EtG antigen was purchased from EastCoast Bio (North Berwick, ME, USA). Antibody stock solution was stored and diluted in 0.15 M 1x PBS (Phosphate buffered Saline) solution. Antigen stock solution was stored in D.I H_2_O (deionized water). Dilutions for the response curve and validation studies were performed in pooled human sweat. Pooled sweat was purchased from Innovative Research (Novi, MI, USA).

### Sensor Fabrication

Figure 1 shows the manufactured sensor platform on polyimide substrate. The chemical structure and step-by-step binding mechanisms is also shown in Fig. 1 as well as the schematic of the chemical structure and binding mechanisms. Also shown is the equivalent circuit for the sensor. The glass and polyimide substrates were consecutively cleaned with 70% isopropyl alcohol (IPA) and de-ionized water for a minute to remove any surface bound impurities. The substrates were then dried under nitrogen prior to fabrication. Patterning was performed using a shadow mask for both the electrode materials. For the Au sensor, the electrode stack comprising a 25 nm Cr layer and 125 nm Au layer was deposited onto the substrates using shadow mask and electron beam cryo-evaporator. In the case of ZnO sensor, the electrode stack comprised of Au sensor stack followed by 125 nm ZnO thin film pattern deposited using shadow mask and radio frequency magnetron sputtering. The optimal ZnO deposition parameters (140 W power, absence of oxygen plasma, 15 mTorr pressure) have been described in our previous work^53^. The thickness of the layers was validated by profilometer measurements. The gold on the ZnO sensor forms an ohmic contact to the ZnO film and the potentiostat. The design ensured that the Au on the ZnO sensor was physically isolated from any functionalization or assay treatment steps for sensing and that the responses were due to the electrochemical interactions on the ZnO.

### Functionalization and Characterization of the sensing surface

Baseline electrical impedance of the fabricated Au and ZnO electrodes on the sensor were evaluated using electrochemical impedance spectroscopy. The sensor surfaces were cleaned with 70% IPA, washed three times, and dried with nitrogen before and after baseline measurements. Electrochemical Impedance Spectroscopy (EIS) measurements were performed at 10 mV peak-to-peak voltage, 10 Hz to quantify the impedance. DSP was dissolved in DMSO to formulate a 10 mM concentration for functionalization on the Au and ZnO sensor surfaces. Successful functionalization of the thiol linker molecules onto electrode sensing areas was validated with impedance measurements pre- and post- DSP treatment. The sensor surface post DSP functionalization was washed with DMSO 3 times to remove any unbound DSP molecules. Following this, we washed the sensor with 0.15 M 1x PBS to neutralize surface pH and prepare for antibody immobilization. For enabling detection of EtG, we immobilized anti-EtG monoclonal antibodies on the electrodes. Monoclonal anti-EtG at a concentration of 10 mg/L was incubated on the sensor surface for 30 minutes at room temperature. SuperBlock^TM^ was used after antibody incubation to seal-off any unbound linker surfaces to avoid non-specific EtG binding directly onto the NHS groups. Impedance measurements at 10 mV, 10 Hz were performed pre and post antibody treatment to validate binding. EtG stock was received at a stock concentration of 1 mg/mL. Serial dilution was first prepared in 0.15 M PBS. The target EtG concentration doses to be tested were diluted from the PBS stock into human sweat at a 1:9 volume ratio.

### Sensor calibration response and continuous monitoring

Target EtG doses spiked in human sweat were tested on the sensor by confining the fluid volume to the sensing region using a waterproof adhesive film as shown in Fig 1. The total volume of test solution introduced on the sensing regions was 3–6 μL. Each EtG target dose was incubated on the sensor for 15 minutes and impedance measurement were performed at 10 mV, 10 Hz. A 3x PBS wash was performed after each dose to remove any unbound molecules from the surface. All doses tested were serially evaluated on the same sensor (intra-assay) as well as on individual sensors (inter-assay) to demonstrate repeatability and robustness for a total of n = 5 replicates. EtG in the concentration range of 1 pg/L–100 μg/L was tested on the ZnO sensor and in the concentration range of 10 ng/L–10 mg/L was tested on the Au sensor. Impedance values were interpreted using a design specific equivalent circuit as shown in Fig 1. Impedance measurements were performed using a Gamry Reference 600 potentiostat (Gamry Instruments, Warminster, PA, USA). Specificity of the signal response was tested by blocking DSP linker -NHS sites with an albumin based blocking buffer followed by attempts with antibody conjugation and dose response testing. Continuous detection of EtG was achieved with a Harvard Apparatus PHD Ultra Syringe Pump (Holliston, MA, USA). Output volume from the pump was set to 1 μL/min and the syringe output was directly placed over the sensing electrode area. Impedance measurements at 10 mV, 10 Hz was performed after 2 minutes of addition of 3 μL of the sample.

## Additional Information

**How to cite this article**: Selvam, A. P. *et al.* A wearable biochemical sensor for monitoring alcohol consumption lifestyle through Ethyl glucuronide (EtG) detection in human sweat. *Sci. Rep.* **6**, 23111; doi: 10.1038/srep23111 (2016).
